# Supplementary material for: Mucosal TLR2-activating protein-based vaccination induces potent pulmonary immunity and protection against SARS-CoV-2 in mice
Source: Nat Commun. 2022 Nov 15;13:6972. doi: 10.1038/s41467-022-34297-3 (PMC9665025; doi:10.1038/s41467-022-34297-3)
Supplement: Supplementary file 1 — Supplementary Information [file 41467_2022_34297_MOESM1_ESM.pdf]

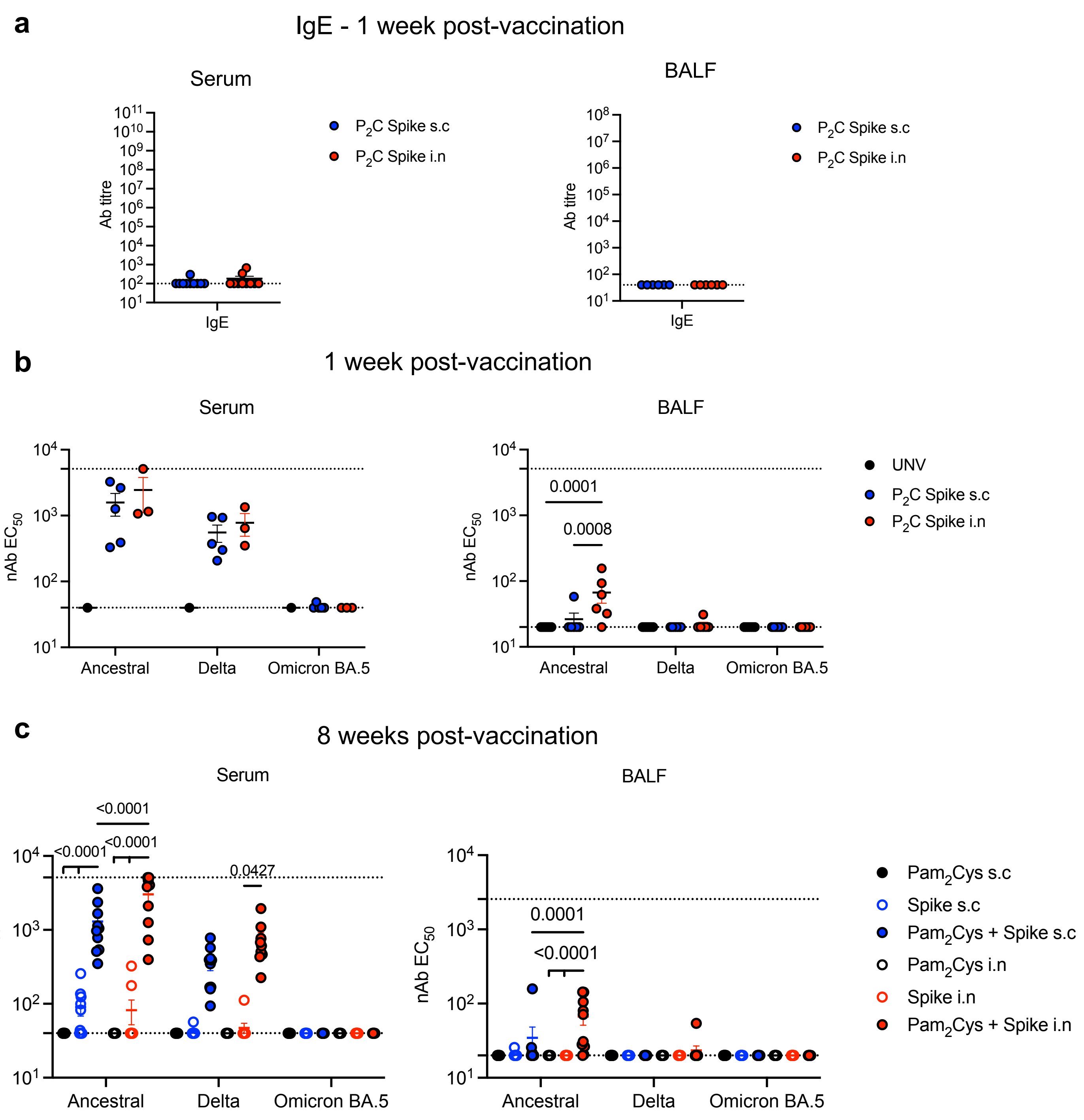

Figure S1. Vaccination with Pam_2Cys (P_2C) Spike induces potent sustained neutralising antibody responses against ancestral and Delta SARS-CoV-2, but not Omicron BA.5. C57BL/6 mice were immunized with Pam_2Cys and Spike protein sub-cutaneously (s.c; blue) or intra-nasally (i.n; red) three times, two weeks apart. (**a**) One week post vaccination, anti-Spike IgE titres ($\log_{10}$ scale) in the serum (n=10 animals) and broncho-alveolar lavage fluid (BALF) (n=6 animals), determined by ELISA, using the mean +3SD of the level in 1:100 dilution of sera or 1:40 BALF from unvaccinated mice as the cut-off. Data points represent individual mice from two independent experiments, means +/- SEM are shown. (**b**) Neutralizing antibody titres (nAb) in the serum and broncho-alveolar lavage fluid (BALF) of vaccinated mice were determined as the titre needed for 50% inhibition of SARS-CoV-2 (variants) infection in HEK293-ACE2-TMPRSS2 cells, at one and (**c**) eight weeks post-final vaccination. Upper and lower limits of detection are indicated by the dotted lines. Data are for individual mice from two independent biological experiments (n=10 animals), means +/- SEM are shown. Statistics: p-values indicated, two-way ANOVA, Tukey's multiple comparisons.

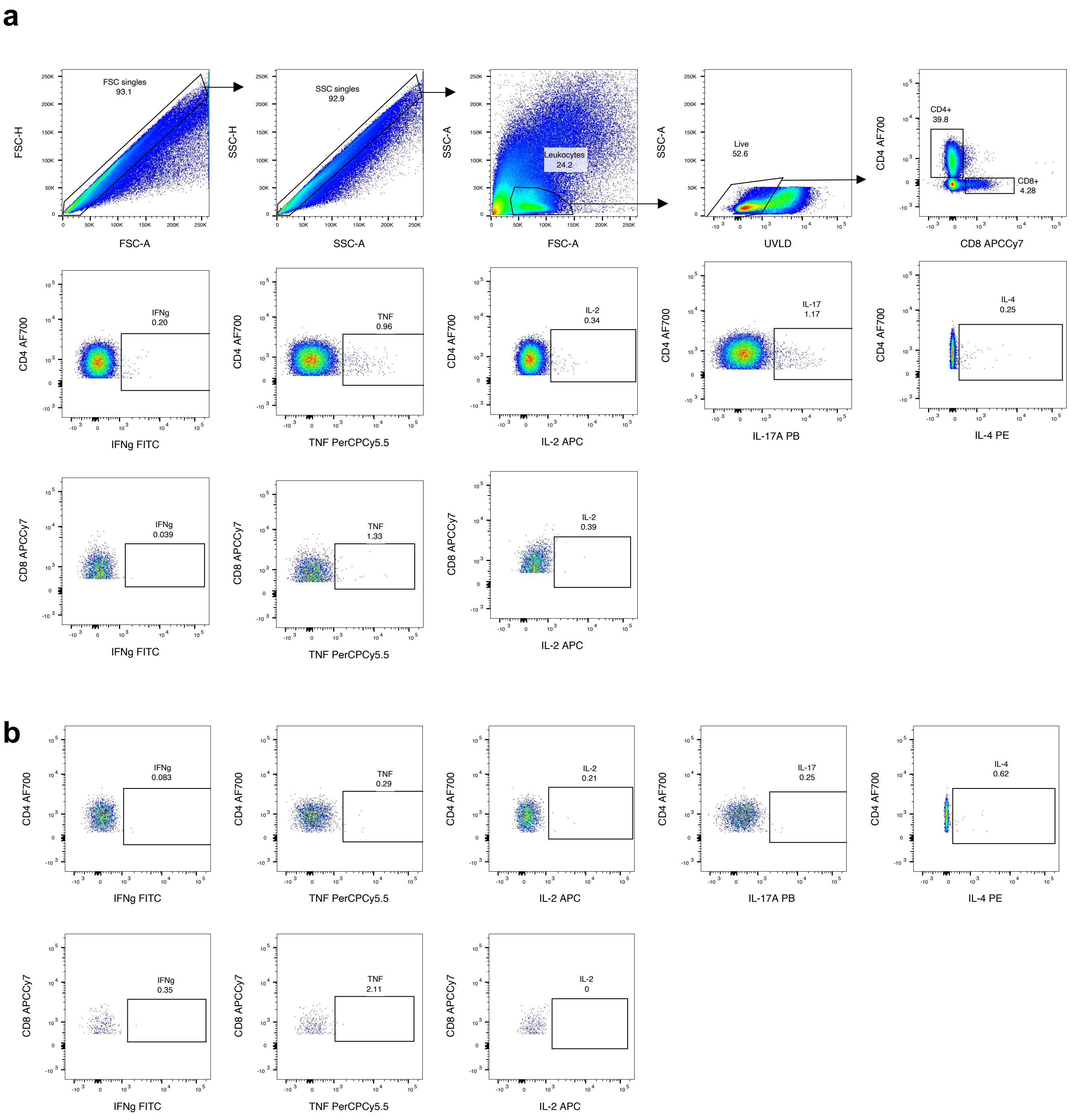

Figure S2. Gating strategy for analysis of flow cytometry data, to quantitate the proportion of Spike-specific cytokine producing T-cells in the lungs or spleen. After quality control gates to exclude doublets and debris, live cells were gated, then CD4$^+$ and CD8$^+$ cells. Cytokine positive cells for each T-cell population were gated. A representative lung sample (from n=12 mice from two independent replicate experiments) is shown (WT mouse reconstituted with *Tlr2-/-* BM, vaccinated intra-nasally with Pam$_2$Cys Spike three times two weeks apart) after *ex vivo* stimulation in the presence of Brefeldin A with (**a**) Spike protein (10 µg/ml) or (**b**) no protein recall, used as a negative control to set gates for cytokine producing cells.

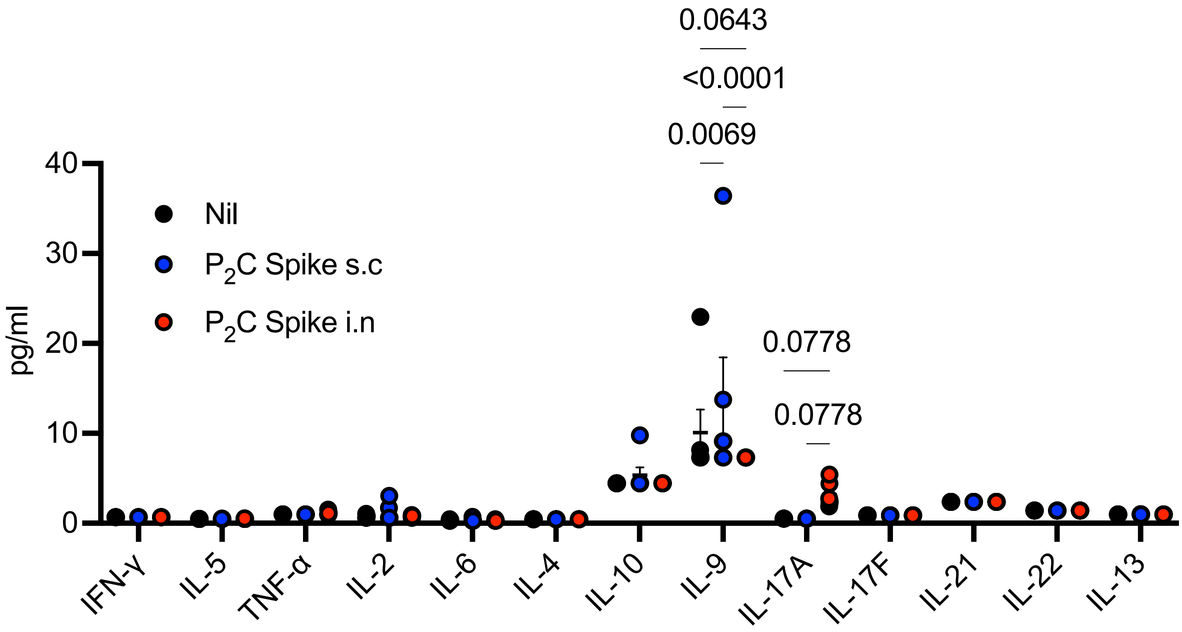

Figure S3. Vaccination with Pam_2Cys Spike induces minimal sustained release of inflammatory cytokines into the airways. C57BL/6 mice were immunized with Pam_2Cys and Spike protein sub-cutaneously (s.c; blue) or intra-nasally (i.n; red) three times, two weeks apart. Th-associated cytokines in the broncho-alveolar lavage fluid (BALF) of vaccinated mice were determined at one week post-final vaccination. Data are for individual mice from two independent biological experiments (n=6 animals), means +/- SEM are shown. Statistics: two-way ANOVA, Tukey's multiple comparisons.

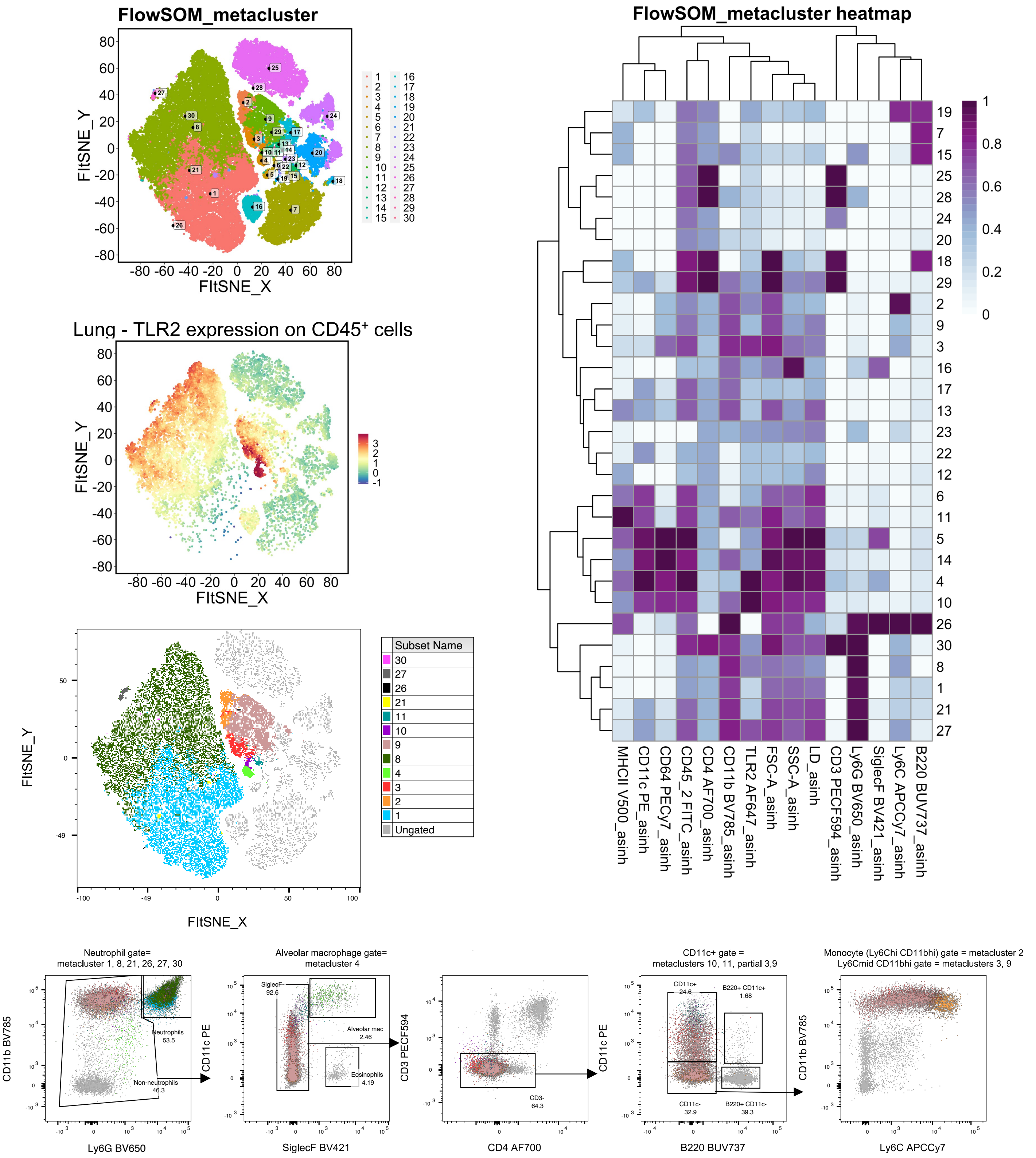

Figure S4. Computational analysis of lung immune cells 24 hours post Pam$_2$Cys Spike intra-nasal. Unsupervised clustering of lung CD45$^+$ cells was performed on flow cytometry data using the Spectre R package. All 30 metaclusters are indicated, alongside their relative expression of surface phenotypic markers. Relative expression of TLR2 across the clusters was examined by FIt-SNE, a representative plot for wild type (WT; BALB/c) mice receiving WT bone marrow is shown. Clusters localizing with upregulated TLR2 expression were subjected to manual gating in FlowJo to confirm cell phenotypes. Clusters 1, 8, 21, 26, 27 and 30 were phenotypically neutrophils (Ly6G$^+$CD11b$^+$; these metaclusters excluded from subsequent gating). Cluster 4, which had the highest degree of TLR2 expression were alveolar macrophages (SiglecF$^+$CD11c^{hi}; excluded from subsequent gating). Metaclusters 10 and 11 which also had high TLR2 were SiglecF$^-$CD11c$^+$ (excluded from subsequent gating), cluster 2 were monocytes (Ly6C^{hi}CD11b^{hi}) and clusters 3 and 9 were CD11c$^{-\text{ to mid}}$Ly6C^{mid}CD11b^{hi}.

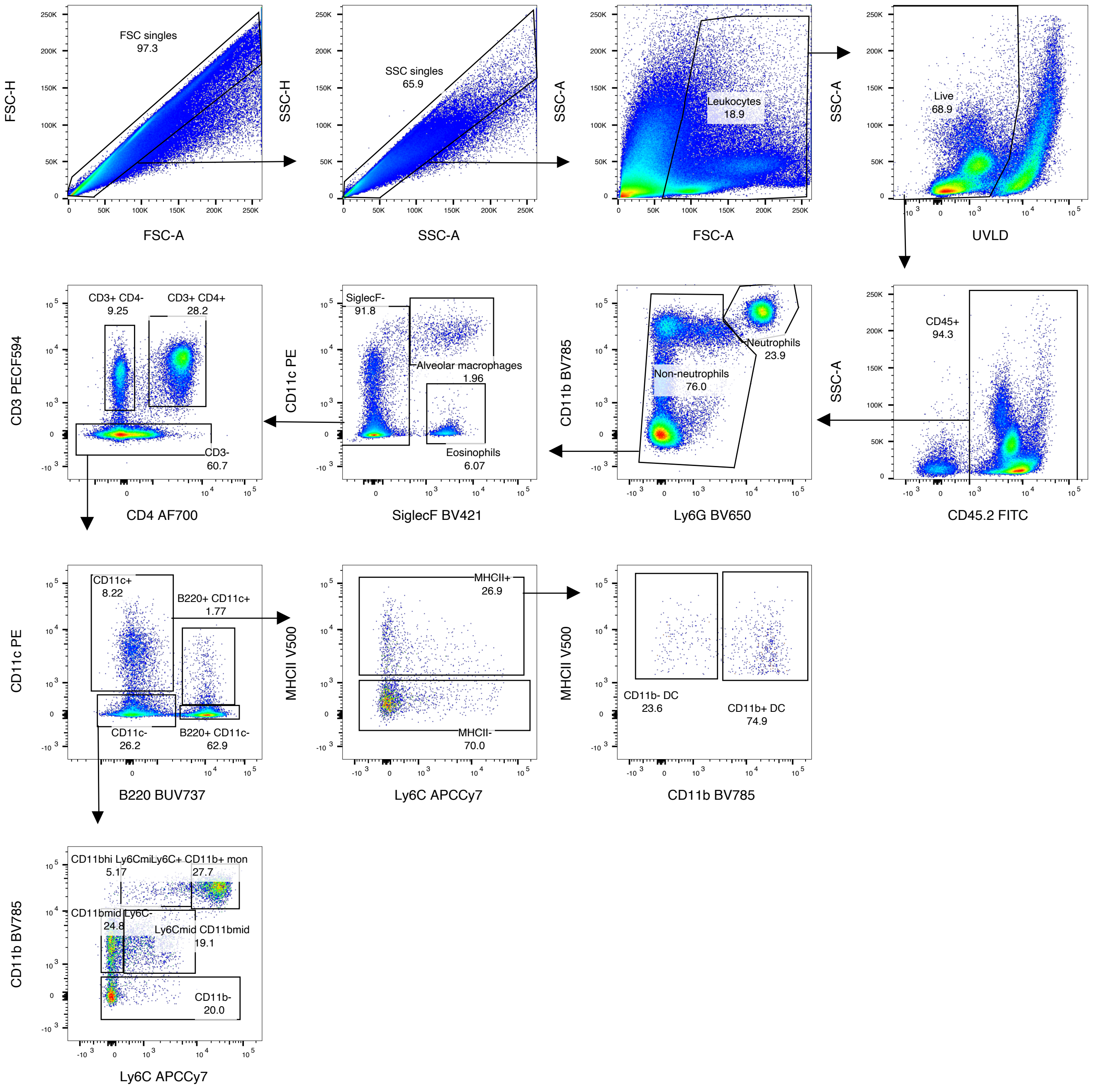

Figure S5. Gating strategy for analysis of flow cytometry data, to characterize lung and broncho-alveolar immune populations described in Figures 5 and S6-8. After quality control gates to exclude doublets and debris, live cells were gated, then CD45^{+} cells. Neutrophils (Ly6G^{+}CD11b^{+}) were gated, then alveolar macrophages (SiglecF^{+}CD11c^{hi}) and eosinophils (SiglecF^{hi}CD11c^{lo}). SiglecF^{-} cells were gated and T-cells defined (CD4^{+}CD3^{-}, CD4^{-}CD3^{+}). CD3^{-} cells were divided into B220^{+}CD11c^{+}, B220^{+}CD11c^{-}, B220^{-}CD11c^{+} and B220^{-}CD11c^{-}. B220^{-}CD11c^{+} cells were divided into MHCII^{-} and MHCII^{+}, then MHCII^{+} cells divided between CD11b^{+} and CD11b^{-}. B220^{-}CD11c^{-} cells were divided into 5 populations based on Ly6C and CD11b expression.

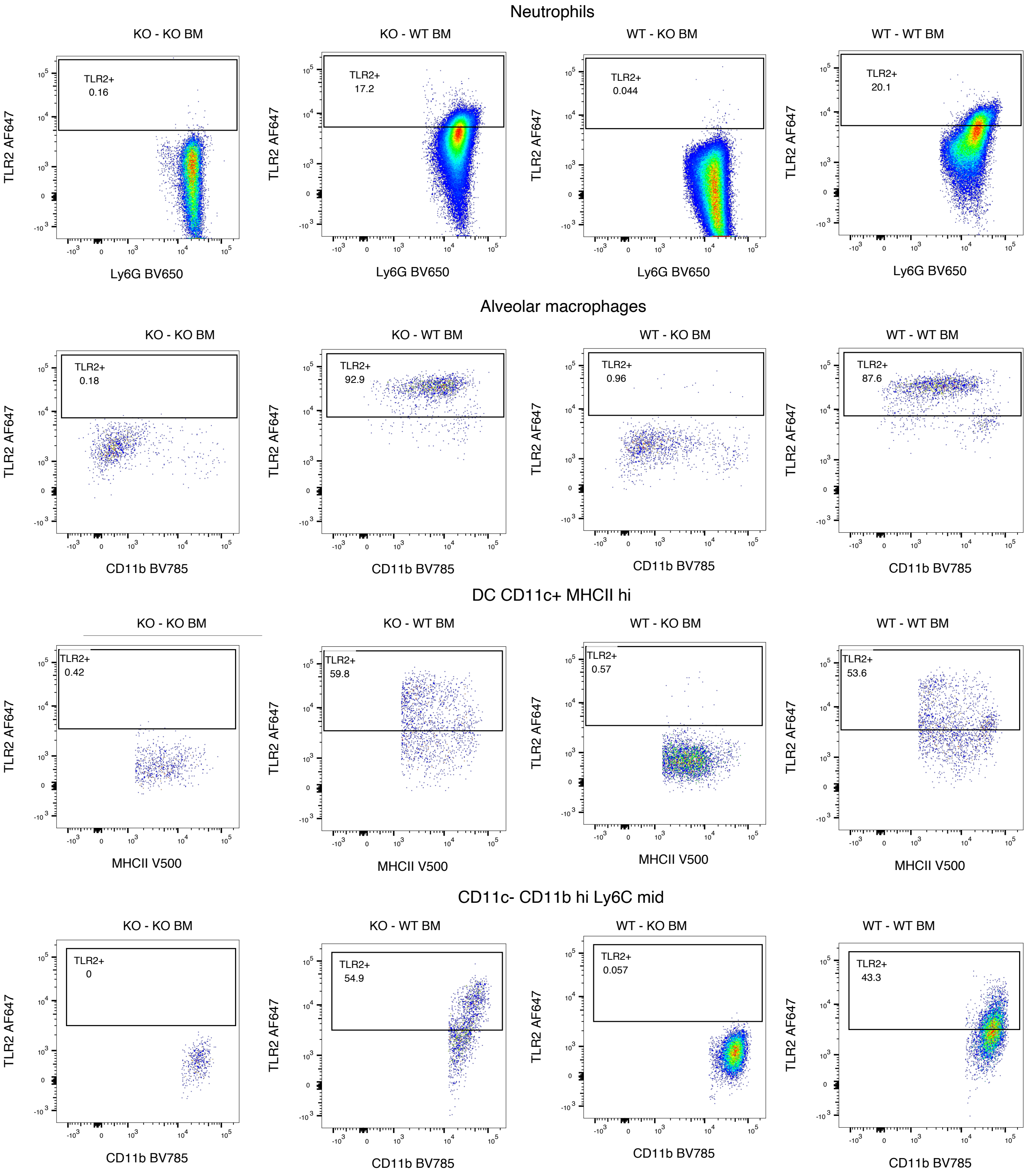

Figure S6. *Tlr2-/-* (KO) or wild type (WT) mice (n=6) were irradiated then received transfer of *Tlr2-/-* or WT bone marrow (BM) cells i.v. Mice were rested for 12 weeks to allow hematopoietic reconstitution and replacement, then were immunized with Pam_2Cys Spike intra-nasally. Immune cells in the lungs were characterized at 24 hours post vaccination by flow cytometry. Expression of TLR2 on key lung populations of interest, a representative sample from each experimental group is shown.

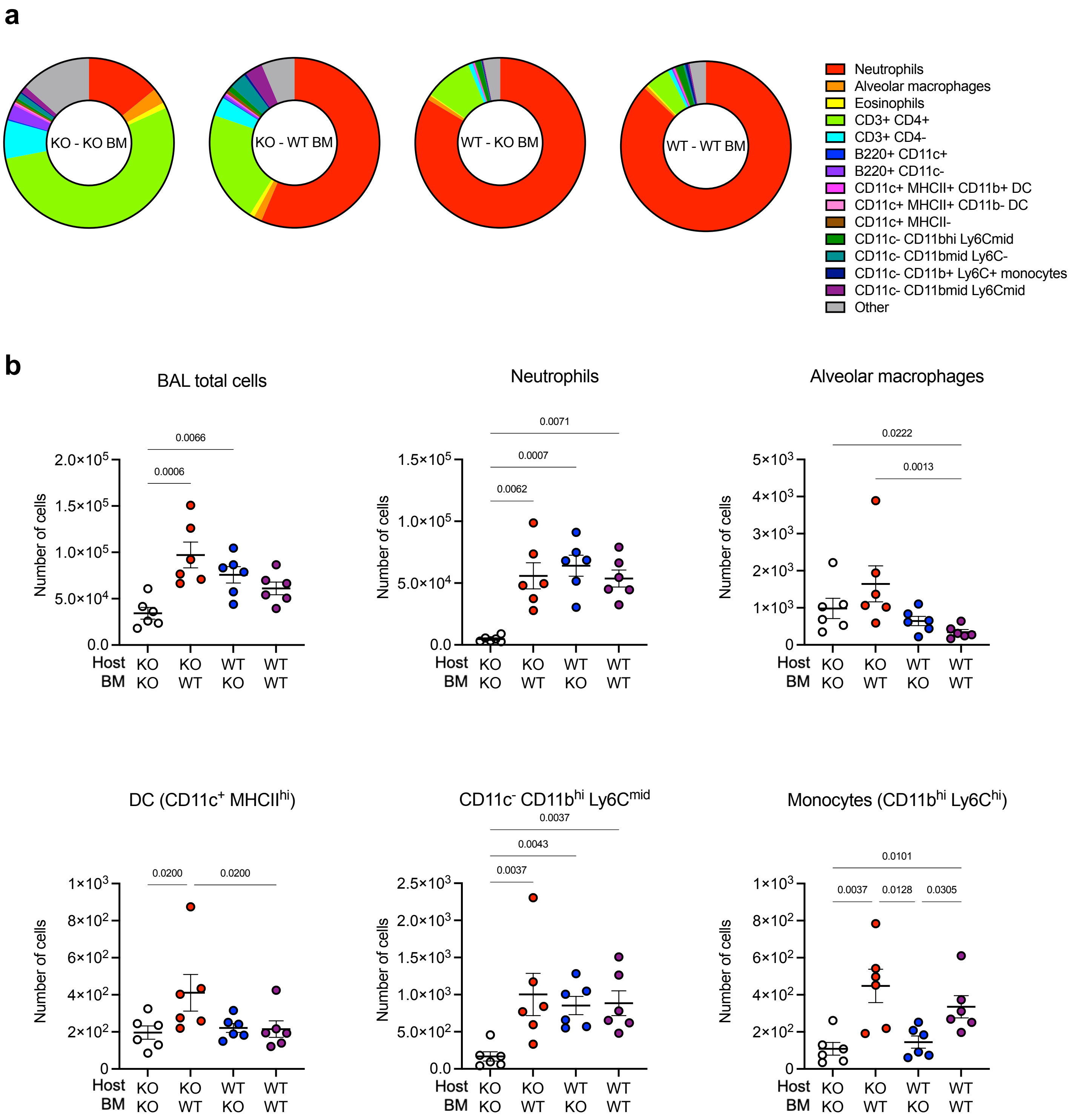

Figure S7. TLR2 expression by both hematopoietic and non-hematopoietic lineages contribute to early innate immune responses in the airways to mucosal vaccination with Pam$_2$Cys Spike. *Tlr2-/-* (KO) or wild type (WT) mice (n=6) were lethally irradiated then received transfer of *Tlr2-/-* or WT bone marrow (BM) cells i.v. Mice were rested for 12 weeks to allow hematopoietic reconstitution and replacement, then immunized with Pam$_2$Cys Spike intra-nasally. Immune cells in the airways (bronchoalveolar lavage; BAL) were characterized at 24 hours post vaccination. (**a**) Mean proportions of CD45$^+$ cell populations in the BAL determined by flow cytometry, with (**b**) quantitation of populations of interest (KO host KO BM, black; KO host WT BM, red; WT host KO BM, blue; WT hose WT BM, purple). Data are for individual mice (n=6), means +/- SEM are shown. Statistics: Kruskal-Wallis test, Dunn's multiple comparisons, p values are indicated.

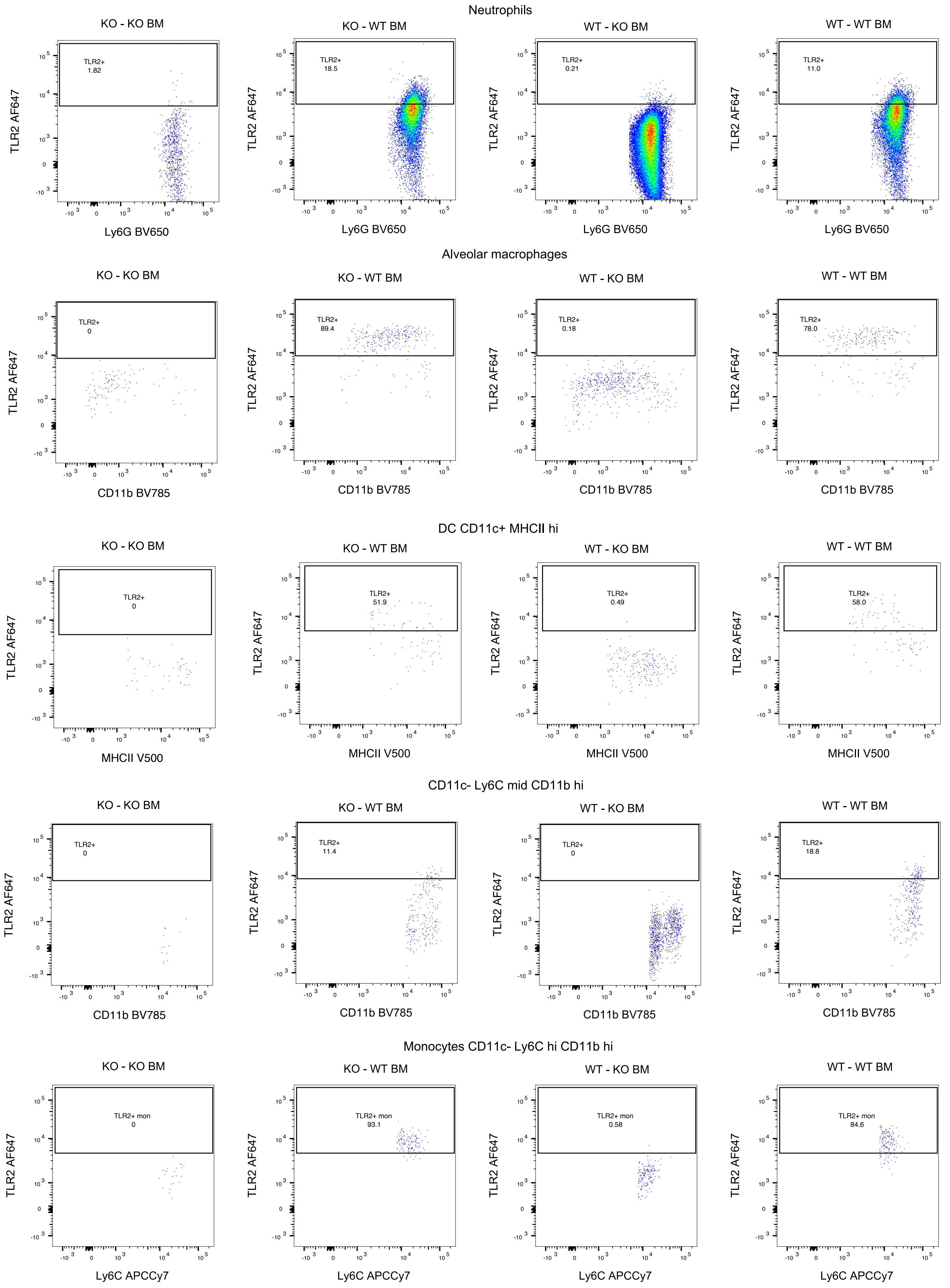

Figure S8. *Tlr2-/-* (KO) or wild type (WT) mice (n=6) were lethally irradiated then received transfer of *Tlr2-/-* or WT bone marrow (BM) cells i.v. Mice were rested for 12 weeks to allow hematopoietic reconstitution and replacement, then were immunized with Pam_2Cys Spike intra-nasally. Immune cells in the airways were characterized at 24 hours post vaccination by flow cytometry. Expression of TLR2 on key airway (bronchoalveolar lavage; BAL) populations of interest, a representative sample from each experimental group is shown.

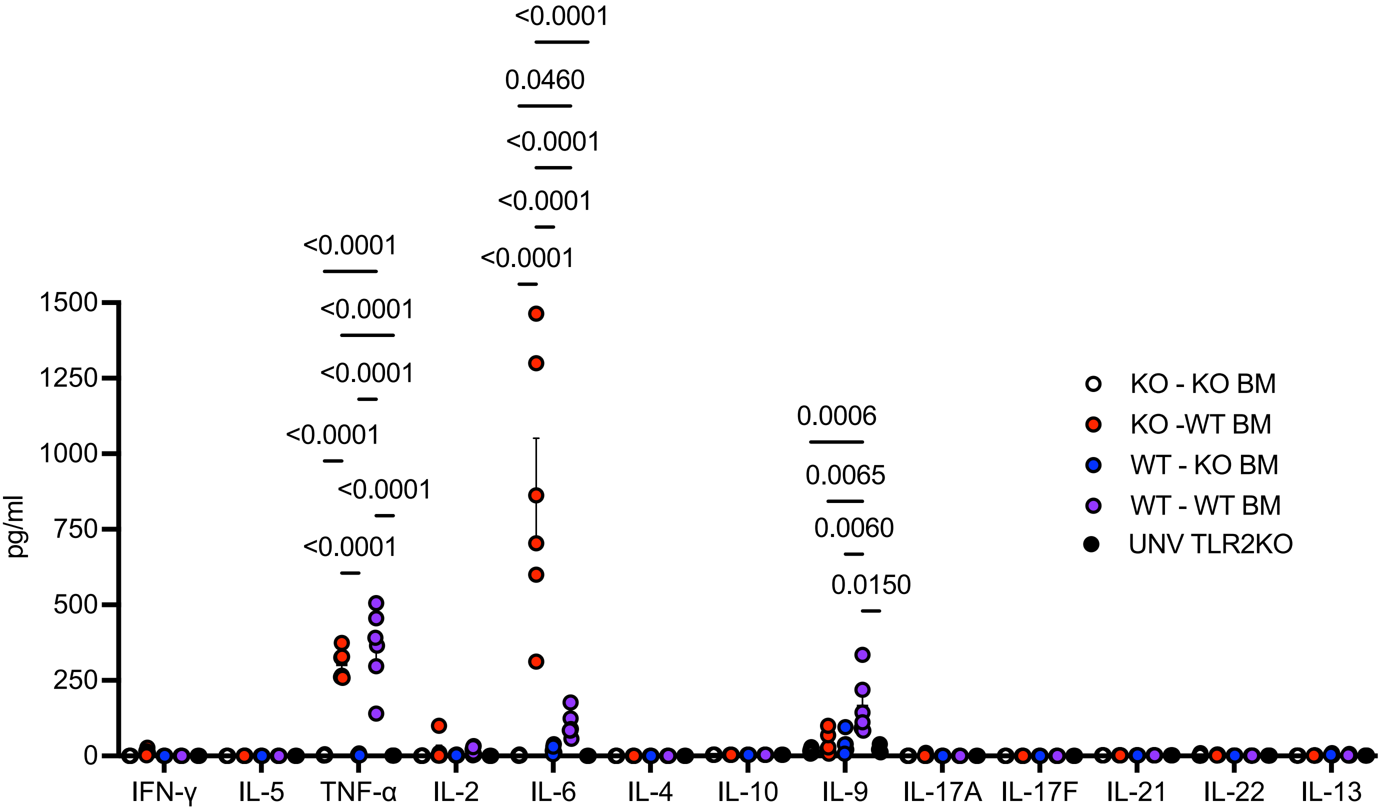

Figure S9. TLR2 expression by hematopoietic cell lineages leads to early cytokine responses in the airways after mucosal vaccination with Pam_2Cys Spike. *Tlr2-/-* (KO) or wild type (WT) mice (n=6) were lethally irradiated then received transfer of *Tlr2-/-* or WT bone marrow (BM) cells i.v. Mice were rested for 12 weeks to allow hematopoietic reconstitution and replacement, then immunized with Pam_2Cys Spike intra-nasally (KO host KO BM, black; KO host WT BM, red; WT host KO BM, blue; WT hose WT BM, purple). Control *Tlr2-/-* mice were left unvaccinated (UNV TLR2KO; black closed circles). Cytokines in the airways (bronchoalveolar lavage fluid; BALF) were characterized at 24 hours post vaccination. Data are for individual mice (n=6), means +/- SEM are shown. Statistics: two-way ANOVA, Tukey's multiple comparisons.

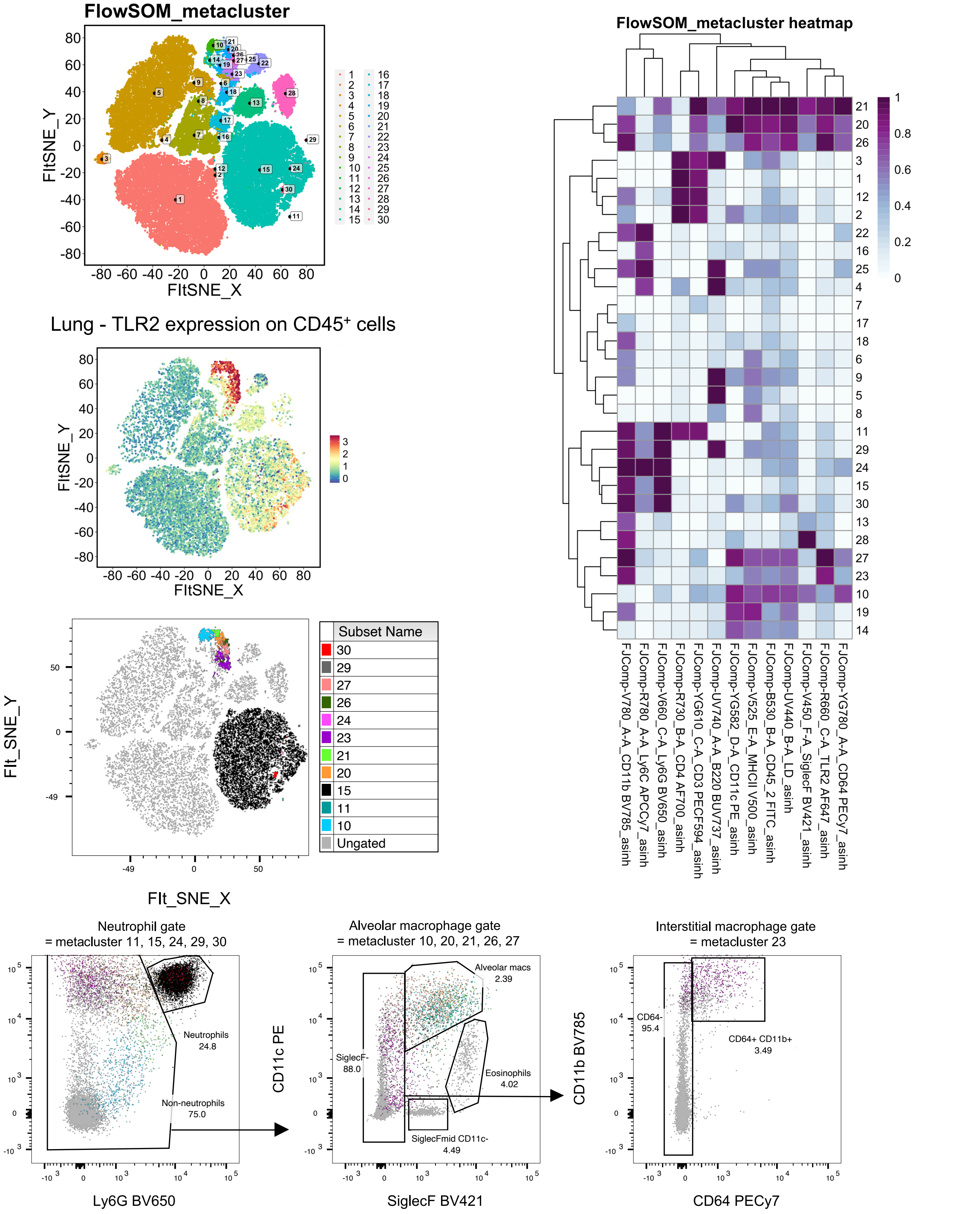

Figure S10. Computational analysis of lung immune cells one week post Pam_2Cys Spike intra-nasal final booster vaccination. Unsupervised clustering of lung $CD45^+$ cells was performed on flow cytometry data using the Spectre R package. All 30 metaclusters are indicated, alongside their relative expression of surface phenotypic markers. Relative expression of TLR2 across the clusters was examined by FIt-SNE, a representative plot for wild type (WT; BALB/c) mice receiving WT bone marrow is shown. Clusters localizing with upregulated TLR2 expression were subjected to manual gating in FlowJo to confirm cell phenotypes. Clusters 11, 15, 24, 29 and 30, which had low to moderate TLR2 expression, were phenotypically neutrophils ($Ly6G^+CD11b^+$; these metaclusters excluded from subsequent gating). Clusters 10, 20, 21, 26 and 27, which localized with high TLR2 expression, were primarily alveolar macrophages ($SiglecF^+CD11c^{hi}$; excluded from subsequent gating) and metacluster 23 were interstitial macrophages ($SiglecF^-CD64^+CD11b^{hi}$).

a

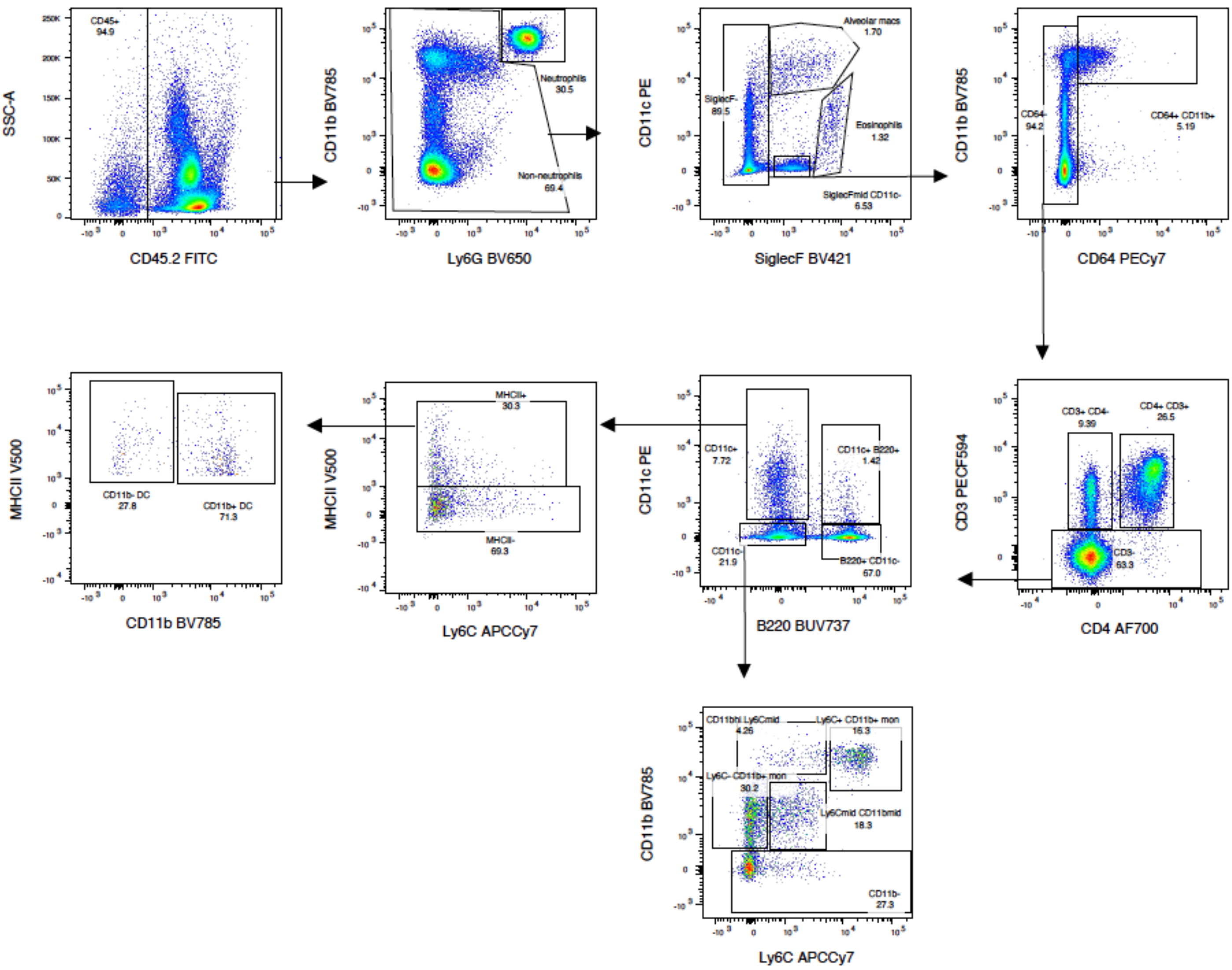

b

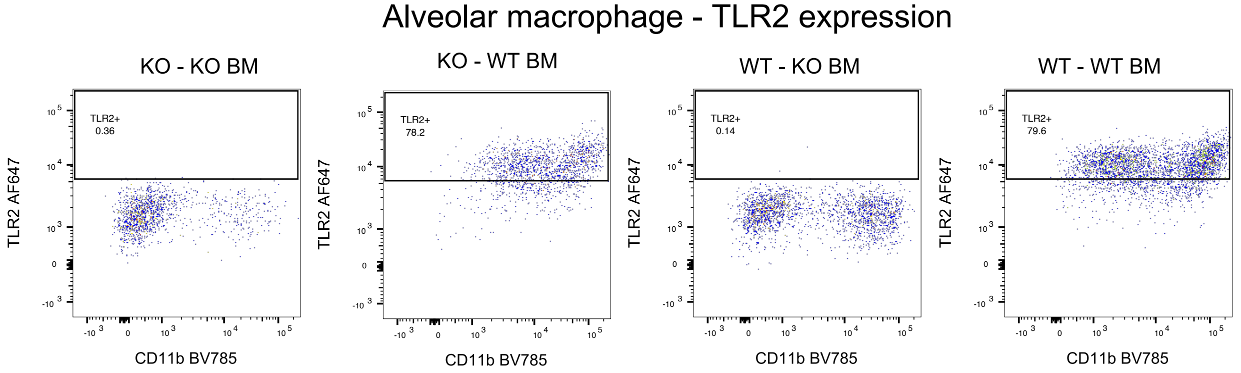

Figure S11. (**a**) Gating strategy for analysis of flow cytometry data, to characterize lung immune populations described in figure 6. After quality control gates to exclude doublets and debris, live cells were gated, then $CD45^{+}$ cells. Neutrophils ($Ly6G^{+}CD11b^{+}$) were gated, then alveolar macrophages ($SiglecF^{+}CD11c^{hi}$), eosinophils ($SiglecF^{hi}CD11c^{lo\text{-}int}$), and $SiglecF^{int}CD11c^{lo}$ cells. $SiglecF^{-}$ cells were gated and divided into interstitial macrophages ($CD64^{+}CD11b^{hi}$) and $CD64^{-}$ cells. T-cells were defined ($CD4^{+}CD3^{-}$, $CD4^{-}CD3^{+}$), then $CD3^{-}$ cells divided into $B220^{+}CD11c^{+}$, $B220^{+}CD11c^{-}$, $B220^{-}CD11c^{+}$ and $B220^{-}CD11c^{-}$. $B220^{-}CD11c^{+}$ cells were divided into $MHCII^{-}$ and $MHCII^{+}$, then $MHCII^{+}$ cells divided between $CD11b^{+}$ and $CD11b^{-}$. $B220^{-}CD11c^{-}$ cells were divided into 5 populations based on Ly6C and CD11b expression. (**b**) Comparison of TLR2 expression on alveolar macrophages, a representative sample from each experimental group (from Figure 6) is shown. *Tlr2-/-* (KO), wild type (WT), bone marrow (BM).

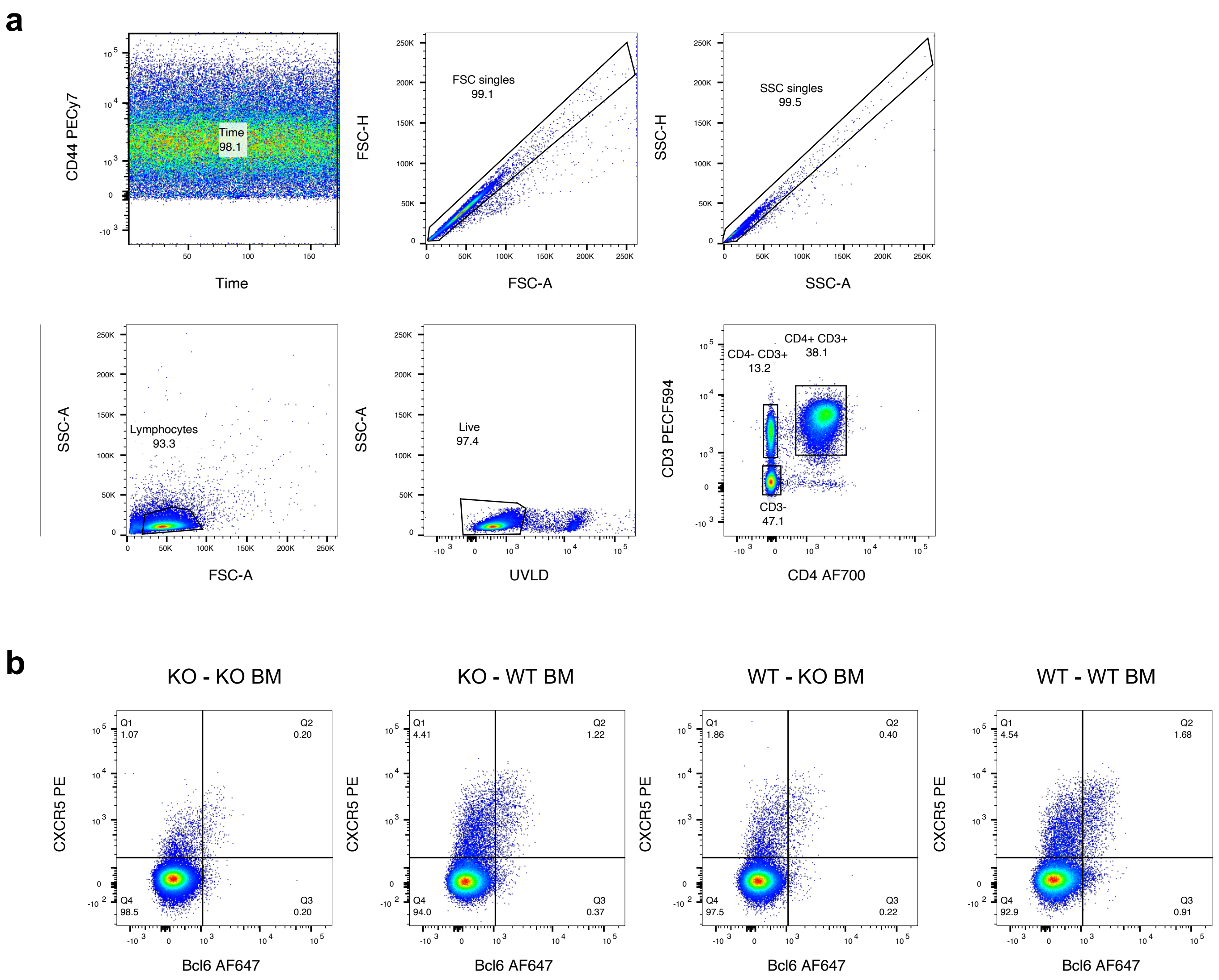

Figure S12. (**a**) Gating strategy for analysis of flow cytometry data, to quantitate the proportion of Bcl6^{+} CD4^{+} T-cells in the mediastinal lymph node of mucosally vaccinated mice. After quality control gates to exclude doublets and debris, live cells were gated, then CD4^{+}CD3^{+} cells. (**b**) Comparison of Bcl6^{+} CD4^{+} T-cells, a representative sample from each experimental group (from Figure 7) is shown. *Tlr2-/-* (KO), wild type (WT), bone marrow (BM).
