# Supplementary material for: Mucosal TLR2-activating protein-based vaccination induces potent pulmonary immunity and protection against SARS-CoV-2 in mice
Source: Nat Commun. 2022 Nov 15;13:6972. doi: 10.1038/s41467-022-34297-3 (PMC9665025; doi:10.1038/s41467-022-34297-3)
Supplement: Supplementary file 3 — Reporting Summary [file 41467_2022_34297_MOESM3_ESM.pdf]

nature portfolio

Corresponding author(s): Warwick J. Britton and Richard J. Payne

Last updated by author(s): Oct 17, 2022

Reporting Summary

Nature Portfolio wishes to improve the reproducibility of the work that we publish. This form provides structure for consistency and transparency in reporting. For further information on Nature Portfolio policies, see our Editorial Policies and the Editorial Policy Checklist.

Statistics

For all statistical analyses, confirm that the following items are present in the figure legend, table legend, main text, or Methods section.

n/a	Confirmed	
☐	☒	The exact sample size (*n*) for each experimental group/condition, given as a discrete number and unit of measurement
☐	☒	A statement on whether measurements were taken from distinct samples or whether the same sample was measured repeatedly
☐	☒	The statistical test(s) used AND whether they are one- or two-sided<br>*Only common tests should be described solely by name; describe more complex techniques in the Methods section.*
☐	☒	A description of all covariates tested
☐	☒	A description of any assumptions or corrections, such as tests of normality and adjustment for multiple comparisons
☐	☒	A full description of the statistical parameters including central tendency (e.g. means) or other basic estimates (e.g. regression coefficient) AND variation (e.g. standard deviation) or associated estimates of uncertainty (e.g. confidence intervals)
☐	☒	For null hypothesis testing, the test statistic (e.g. *F*, *t*, *r*) with confidence intervals, effect sizes, degrees of freedom and *P* value noted<br>*Give P values as exact values whenever suitable.*
☒	☐	For Bayesian analysis, information on the choice of priors and Markov chain Monte Carlo settings
☒	☐	For hierarchical and complex designs, identification of the appropriate level for tests and full reporting of outcomes
☒	☐	Estimates of effect sizes (e.g. Cohen's *d*, Pearson's *r*), indicating how they were calculated

Our web collection on statistics for biologists contains articles on many of the points above.

Software and code

Policy information about availability of computer code

Data collection	Flow cytometry - BD FACSDiva v8.0.3. Imaging - Harmony® high-content analysis software v4.9 (Perkin Elmer), InCarta software v4.6 (Cytiva, USA)
Data analysis	Statistics: GraphPad Prism v9. Flow cytometry: FlowJo v10 (BD), Spectre R package v1.0 (instructions and all source code provided at https://github.com/ImmuneDynamics/spectre), uses FlowSOM algorithm v1.5.4 and FIt-SNE algorithm v 1.2.1.

For manuscripts utilizing custom algorithms or software that are central to the research but not yet described in published literature, software must be made available to editors and reviewers. We strongly encourage code deposition in a community repository (e.g. GitHub). See the Nature Portfolio guidelines for submitting code & software for further information.

Data

Policy information about availability of data

All manuscripts must include a data availability statement. This statement should provide the following information, where applicable:

- Accession codes, unique identifiers, or web links for publicly available datasets
- A description of any restrictions on data availability
- For clinical datasets or third party data, please ensure that the statement adheres to our policy

Source data are provided with this paper. The full datasets and raw data files generated during and/or analysed in the current study are available from the corresponding author on reasonable request.

Field-specific reporting

Please select the one below that is the best fit for your research. If you are not sure, read the appropriate sections before making your selection.

- [x] Life sciences
- [ ] Behavioural & social sciences
- [ ] Ecological, evolutionary & environmental sciences

For a reference copy of the document with all sections, see nature.com/documents/nr-reporting-summary-flat.pdf

Life sciences study design

All studies must disclose on these points even when the disclosure is negative.

Sample size	A minimum of n=3 and maximum of n=6 mice per experimental group were utilised, in order to provide minimum numbers for calculation of standard error of the mean in each individual experiment. Sample size was determined using the resource equation method, the degree of freedom of analysis of variance (ANOVA) "E" is calculated using the equation: E = Total number of animals − Total number of groups, E between 10 and 20 is considered adequate
Data exclusions	No data were excluded, unless there was a known technical error during sample processing (e.g handling error of sample).
Replication	Detailed in figure legends - in most cases, data is pooled from two independent biological replicate experiments, or is representative of two independent biological replicate experiments. Individual data points are shown in the figures.
Randomization	Age-matched mice or cells were randomly allocated to experimental groups.
Blinding	Investigators were not blinded during data acquisition or analysis due to limited investigator access and availability. Due to COVID-19 restrictions and facility access restrictions, it was not possible to have sufficient investigator numbers to perform animal experiments in a blinded manner.

Reporting for specific materials, systems and methods

We require information from authors about some types of materials, experimental systems and methods used in many studies. Here, indicate whether each material, system or method listed is relevant to your study. If you are not sure if a list item applies to your research, read the appropriate section before selecting a response.

Materials & experimental systems

n/a	Involved in the study	
☐	☒	Antibodies
☐	☒	Eukaryotic cell lines
☒	☐	Palaeontology and archaeology
☐	☒	Animals and other organisms
☒	☐	Human research participants
☒	☐	Clinical data
☒	☐	Dual use research of concern

Methods

n/a	Involved in the study	
☒	☐	ChIP-seq
☐	☒	Flow cytometry
☒	☐	MRI-based neuroimaging

Antibodies

Antibodies used

All anti-mouse. Data provided are specificity-flurophore (clone; source company; catalogue number; lot number).
CD16/CD32 (2.4G2; BD Biosciences; 553142; 7248907, 0148675), B220-BUV737 (RA3-6B2; BD; 612838; 8225738), SiglecF-BV421 (E50-2440; BD; 562681; 9316255), MHCII-V500 (M5/114.15.2; BD; 562366; 9210432), Ly6G-BV650 (1A8; Biolegend; 127641; B260844), CD11b-BV785 (M1/70; Biolegend; 101243; B305760), CD45.2-FITC (104; BD; 553772; 5027587), CD11c-PE (N418; Biolegend; 117307; B154997), CD3-PECF594 (145-2C11; BD; 562286; 0066150), CD64-PECy7 (X54-5/7.1; Biolegend; 139314; B247012), TLR2-AF647 (6C2; BD; 562625; 9350581), CD4-AF700 (RM4-5; Biolegend; 100536; B248742, B337974) or CD4-AF700 (GK1.5; eBioscience; 56-0041-82; E08943-1633), Ly6C-APCCy7 (HK1.4; Biolegend; 128026; B275668), anti-CXCR5-biotin (2G8; BD; 551960; 0160485), anti-mouse PD1-BV785 (29F1A12; Biolegend; 135225; B337077), CD44-PECy7 (IM7; BD; 560569; 0100100), CD8-APCCy7 (53-6.7; BD; 557654; 8319924), Bcl6-AF647 (K112-91; BD; 561525; 1067317), CD3 (1452C11; 5 μg/mL; in house) and anti-mouse CD28 (37.51; 5 μg/mL; BD Pharmingen), IFNγ-FITC (XMG1.2; BD Pharmingen; 554411; 9197282), IL-17A-PB (TC11-18H10.1; Biolegend; 506918; B230225), IL-4-PE (11B11; BD; 554435; 9319911), TNF-PerCPCy5.5 (MP6-XT22; BD; 560659; 0227403), and IL-2-APC (JES6-5H4; Biolegend; 503810; B281632), CD62L-BV421 (MEL-14; BD; 562910; 5339687 or 9189629), CD103-FITC (2E7; Biolegend; 121419; B274032), CD44-PE (IM7; Biolegend; 103008; B334370), CD69-PECy7 (H1.2F3; BD; 552879; 9098844), CD45.2-APC (104; BD; 558702; 5092510)
For ELISA all Horseradish peroxidase-conjugated: goat anti-mouse IgG (Invitrogen Novex, cat# A16090, lot# 22-176-020112, used at 1:2000), goat anti-mouse IgG1 (Jackson ImmunoResearch cat#115-035-205, lot# 113095, used at 1:2000) goat anti-mouse IgG2c (Jackson ImmunoResearch cat#115-035-208, lot# 108086, used at 1:2000), goat anti-mouse IgA (Invitrogen, cat# 626720, lot# 1490901A, used at 1:2000), rat anti-mouse IgE (monoclonal 23G3; Abcam, cat# ab99574, lot# GR3412160-1, used at 1:2000).

Validation	See manufacturer's websites for details of validation. BD antibodies- manufacturer reports species reactivity as "QC Testing: Mouse", application notes "Flow cytometry: Routinely Tested". Biolegend antibodies - manufacturer reports species reactivity as "Verified Reactivity: Mouse" and application notes "Flow Cytometry - Quality tested". eBioscience - manufacturer reports "This Antibody was verified by Relative expression to ensure that the antibody binds to the antigen stated." Application testing performed for flow cytometry. Invitrogen: reports antibody application testing dilutions for ELISA, and species reactivity with mouse. Jackson ImmunoResearch: reports antibody application testing dilutions for ELISA, and species reactivity with mouse. Abcam: reports species specificity for mouse and tested application for ELISA.

Eukaryotic cell lines

Policy information about cell lines

Cell line source(s)	HEK293T (ATCC CRL-3216) and VeroE6 (ATCC CRL-1586). HEK-ACE2-TMPRSS2 - generated by the investigators, described in Ashhurst et al, J Med Chem, 2021, DOI: 10.1021/acs.jmedchem.1c01494
Authentication	Cell lines were not authenticated for this study.
Mycoplasma contamination	Cell lines were routinely checked for mycoplasma contamination (MycoAlert™ PLUS Mycoplasma Detection Kit; Lonza) and confirmed as negative.
Commonly misidentified lines (See ICLAC register)	No commonly misidentified cell lines were used in the study.

Animals and other organisms

Policy information about studies involving animals; ARRIVE guidelines recommended for reporting animal research

Laboratory animals	Mice - all female. Ages at start of experiment indicated. C57BL/6 (6-8 weeks), BALB/c (6-17 weeks), Tlr2-/- (BALB/c background; 6-17 weeks), K18-hACE2 (8-12 weeks). All mice were housed under strict husbandry conditions including the use of IVC cages and sterilised bedding, food and water. Mice were on a 12 hour light, 12 hour dark cycle, temperature maintained at approximately 21°C and a relative humidity of 45-46%. The light dark cycle, temperature and relative humidity were all monitored by computer. Mice were provided with tissues for nesting material and sterile cardboard and shelter for environment enrichment.
Wild animals	The study did not involve wild animals.
Field-collected samples	The study did not use field-collected samples.
Ethics oversight	All animal experiments were conducted in full compliance with local and institutional guidelines, with approvals from the Sydney Local Health District Animal Welfare Committee (2020-003, 2020-019). These adhere to the Australian Code for the Care and Use of Animals for Scientific Purposes (2021) as set out by the National Health and Medical Research Council of Australia.

Note that full information on the approval of the study protocol must also be provided in the manuscript.

Flow Cytometry

Plots

Confirm that:

- [x] The axis labels state the marker and fluorochrome used (e.g. CD4-FITC).
- [x] The axis scales are clearly visible. Include numbers along axes only for bottom left plot of group (a 'group' is an analysis of identical markers).
- [x] All plots are contour plots with outliers or pseudocolor plots.
- [x] A numerical value for number of cells or percentage (with statistics) is provided.

Methodology

Sample preparation	Murine lung tissue, lymph node, spleen and cells from bronchoalveolar lavage - sample processing is detailed in manuscript methods.
Instrument	BD LSRII 5L or Fortessa X20 flow analyzer
Software	BD FACSDiva (acquisition only, v8.0.3), FlowJo v10 (BD), Spectre R package (v1.0, instructions and source code provided at https://github.com/ImmuneDynamics/spectre)
Cell population abundance	Cell population abundance was determined via analysis with FlowJo v10, gating strategies and population phenotypes are detailed in the manuscript and supplementary information. For quantitation, total leukocyte counts per sample were performed by hemocytometer with trypan blue exclusion.
Gating strategy	Gating strategies used for each experimental antibody panel are detailed in the supplementary information.

- [x] Tick this box to confirm that a figure exemplifying the gating strategy is provided in the Supplementary Information.
